# A retrospective multi-site examination of chronic kidney disease using longitudinal laboratory results and metadata to identify clinical and financial risk

**DOI:** 10.1186/s12882-024-03869-4

**Published:** 2024-12-06

**Authors:** Mark Fung, Aya Haghamad, Elizabeth Montgomery, Kathleen Swanson, Myra L. Wilkerson, Kimon Stathakos, Richard VanNess, Sarah A. Nowak, Clayton Wilburn, Haluk Kavus, Mohammed Amer Swid, Nkemakonam Okoye, Yonah C. Ziemba, Girish Ramrattan, Jonathan Macy, John McConnell, Mary Jane Lewis, Beth Bailey, Khosrow Shotorbani, James M. Crawford

**Affiliations:** 1 Department of Pathology and Laboratory Medicine, University of Vermont College of Medicine, Burlington, VT USA; 2 Department of Pathology and Laboratory Medicine, Northwell Health, New Hyde Park, NY USA; 3 National Kidney Foundation, New York, NY USA; 4 Project Santa Fe Foundation, Salt Lake City, UT USA; 5 Department of Pathology and Laboratory Medicine, Geisinger Medical Center, Danville, PA USA; 6 TriCore Reference Laboratories, Albuquerque, NM USA

**Keywords:** Diabetes, Heart failure, Risk adjustment, Clinical lab 2.0, Clinical laboratory, Population health, Project Santa Fe

## Abstract

**Background:**

A retrospective observational study was conducted at 3 health care organizations to identify clinical gaps in care for patients with stage 3 or 4 chronic kidney disease (CKD), and financial opportunity from U.S. risk adjustment payment systems. Lack of evaluation for CKD in patients with diabetes was also assessed.

**Methods:**

Outpatient longitudinal laboratory results and patient metadata available in the electronic medical record, laboratory information system, and/or laboratory billing or facility claims data for the calendar year 2021 were evaluated. Laboratory results were compared to billing data (ICD-10 codes) and risk adjustment scores including Hierarchical Condition Categories (HCC) to determine if laboratory-identified CKD was coded as a disease condition in the electronic medical record. Adults 18 to 75 years of age were included; inpatient laboratory results and pregnant individuals were excluded.

**Results:**

At the 3 institutions, 12,478 of 16,063 (78%), 487 of 1511 (32%) and 19,433 of 29,277 (66%) of patients with laboratory evidence of stage 3 or 4 CKD did not have a corresponding ICD-10 or HCC code for CKD in the electronic medical record. For patients at the 3 institutions with diabetes on the basis of an HbA1c value of ≥ 6.5%, 34,384 of 58,278 (59%), 2274 of 2740 (83%) and 40,378 of 52,440 (77%) had not undergone guideline-recommended laboratory testing for CKD during the same 12 months. Using publicly available data for calendar year 2021, an estimated 3246 of 32,398 patients (9.9%) at the 3 institutions with undocumented CKD stages 3–4 would be enrolled in Medicare Advantage or Affordable Care Act Marketplace programs. The imputed lost reimbursement under risk-adjusted payment systems for under-documentation of CKD in this subset of patients was $2.85 M for the three institutions combined, representing lost opportunity for both identification and proactive clinical management of these patients, and financial recovery for the costs of providing that care.

**Conclusions:**

Clinical laboratories can provide value beyond routine diagnostics, helping to close gaps in care for identification and management of CKD, stratifying subgroups of patients to identify risk, and capturing missed reimbursement through risk adjustment factors.

**Supplementary Information:**

The online version contains supplementary material available at 10.1186/s12882-024-03869-4.

## Background

Chronic kidney disease (CKD) remains a largely under-recognized and growing public health issue. Of the estimated 37 million U.S. adults with CKD, for individuals not under the care of a nephrology practice approximately 90% remain unaware of their condition [1, 2]. The leading causes of CKD in the United States are diabetes and heart disease, accounting for every 3 out of 4 new cases [3]. The incidence of CKD is on the rise due to the increase in prevalence of risk factors including diabetes, hypertension and an aging population [4]. Conversely, CKD is a strong risk factor for the adverse outcomes of heart failure and its accompanying mortality [5, 6], and for markedly increased health care costs. At the national level, in 2019 the U.S. economic impact of treating Medicare beneficiaries with CKD totaled $87 billion [7].

Early identification of CKD in the ambulatory primary care setting enables interventions which may reduce disease progression and accompanying morbidity and mortality. Best-practice guidelines from the Kidney Disease: Improving Global Outcomes (KDIGO, [8]) organization include improved glycemic control in patients with diabetes and management of hypertension, which have a beneficial effect on the progression of nephropathy [9]. New strategies have also emerged to improve outcomes for diabetic kidney disease [10, 11]. Effective management of diabetes and hypertension in patients with CKD also lowers health care resource utilization and total costs of delivering care [12]. Delay in CKD identification exacerbates CKD as a disease multiplier, as it is associated with progression in cardiovascular morbidity and with premature cardiovascular mortality [13, 14]. Indeed, nearly 50% of patients with CKD die from cardiovascular disease (CVD) before developing end-stage renal disease [13].

Underdiagnosis and missed opportunity for intervention in care of patients with CKD has important implications for health care under the value-based payment system now being advanced in the U.S. Identification of individuals with decreased kidney function through measurement of serum creatinine and calculation of estimated glomerular filtration rate (eGFR), and screening of patients with diabetes for urine albumin:creatinine ratio (ACR) are central to risk management of this population [15]. These two measures are central to the National Committee for Quality Assurance (NCQA) “Kidney Health Evaluation for Patients with Diabetes” Healthcare Effectiveness Data and Information Set (HEDIS) measure [16, 17].

Under the U.S. value-based payment systems, another aspect of the financial impact of CKD is in the form of risk adjustment of reimbursement payments for health care services. In the first instance, primary identification of CKD on the basis of eGFR screening identifies patients at risk for increased total cost of health care. Screening of patients with diabetes for CKD further enhances the opportunity for proactive identification of patients at risk and intervention in the potential progression of their kidney disease [15]. In the second instance, failure to identify patients with CKD (without-or-with comorbid conditions) puts a health care organization at risk both for incurring costs for a patient who is not being properly managed for CKD, and for lost reimbursement opportunity under value-based payment plans, owing to the organization’s failure to properly document and thereby risk adjust these patients [18]. Better patient care and improved documentation and risk adjustment also impact other U.S. quality ratings for health care providers, such as Center for Medicaid and Medicare Services (CMS) quality ratings under the HEDIS system, and the opportunity for shared savings through Accountable Care Organizations [19]. Lastly, suboptimal patient outcomes against industry standards may lead payers to refer patients to other health care providers who have better quality measures [20].

The clinical laboratory, with its testing for and provision of quantitative data to monitor patient CKD status longitudinally, is uniquely positioned to play a crucial role in the early identification and risk stratification of patients with this often asymptomatic and underdiagnosed disease. In so doing, the clinical laboratory also can play a key role in promoting improved population health, inclusive of participating in programmatic opportunities at the population level. This concept of the expanded role of the laboratory in population health has been articulated as Clinical Lab 2.0 by the Project Santa Fe Foundation [21], and was the inspiration for initiating this study.

Our hypothesis was that rigorous examination of existing laboratory data in the laboratory information system or electronic medical record would identify previously unknown patients with CKD, who are eligible for health care that was not yet being provided. Our goal was to gain insight into both the potential clinical and financial impact of these previously unidentified patients, under a value-based payment paradigm. We included financial analysis in this study, because the U.S. Centers for Medicare & Medicaid Services (CMS) is specifically using value-based payments to drive better health care delivery for beneficiaries of CMS programs [22]. We examined data from three integrated health systems in the U.S., each with extensive primary care ambulatory networks that would be able to refer patients to subspecialist providers such as Nephrology for continuity in care when clinically indicated. In our analysis of de-identified data from these health systems, we affirm that underdiagnosis of CKD is a major issue. Our data provide further argument for the compelling need to improve early identification of this chronic condition.

## Methods

A retrospective, observational non-interventional study was conducted at three Project Santa Fe Foundation member organizations, in collaboration with the National Kidney Foundation, to identify clinical gaps in care, and financial opportunity based on the U.S. risk adjustment payment system for CKD. Outpatient longitudinal laboratory results and patient metadata available in the electronic medical record, laboratory information system, and/or laboratory billing or facility claims data for the calendar year 2021 were evaluated. Patients meeting guidelines for laboratory diagnostic criteria of CKD, diabetes, and/or heart failure were included (see below). Laboratory results for patients with CKD were compared to billing data (ICD-10 codes) and risk scores including Hierarchical Condition Categories (HCC) and Johns Hopkins risk score data to achieve the study’s objectives.

### Clinical and administrative inclusion criteria

The study included adults 18 to 75 years of age with outpatient laboratory results indicating a diagnosis of CKD Stage 3 or 4, and/or on the basis of ICD-10 or HCC coding for CKD Stage 3 or 4, for patient records in the laboratory information system and/or electronic medical record from January 1 to December 31, 2021. If laboratory data identified CKD Stage 3 or 4, we searched for similar laboratory testing at least 3 months prior, in fulfillment of KDIGO criteria for diagnosis of CKD [8]. This included “look back” into calendar year 2020 for patients with laboratory testing performed in the first 3 months of 2021. We did not “look forward” into calendar year 2022 to see if there was confirmatory laboratory testing that followed our index laboratory results, nor did we evaluate whether there was progression (or not) of the eGFR between the two measurements. Individuals under the age of 18 or those above the age of 75 years of age, and pregnant individuals were excluded, as were laboratory results from the inpatient setting. The study excluded patients with CKD Stage 1, Stage 2 and Stage 5.

**Fig. 1:**
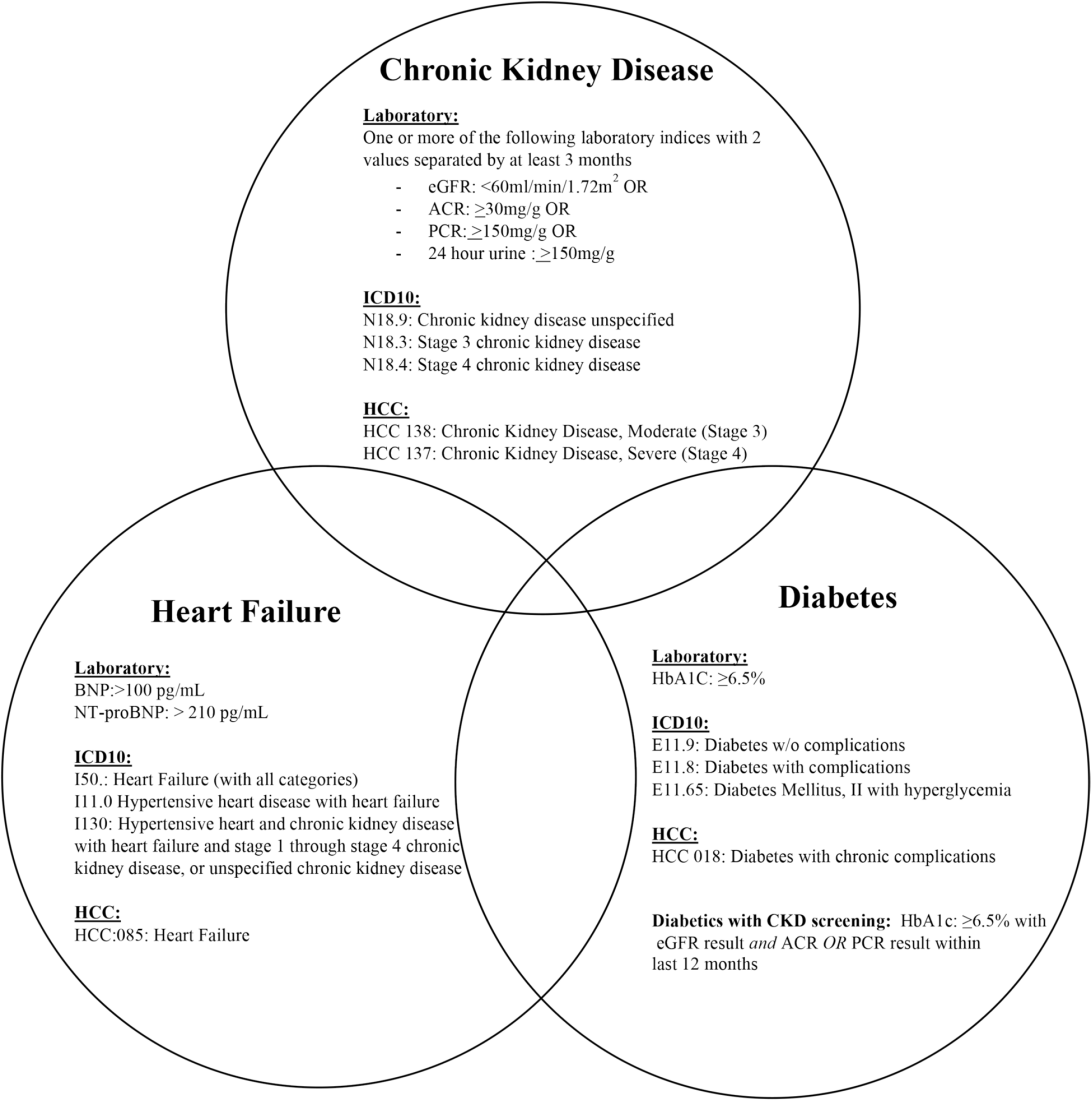
Laboratory definitions, ICD-10 codes, and HCC codes for chronic kidney disease, diabetes, and heart failure. In this study, patients with CKD were identified by laboratory data and/or ICD-10 or HCC codes for the purposes of study objectives regarding fidelity of diagnostic coding for CKD; patients with diabetes and heart failure were identified on the basis of laboratory data alone. ACR, urine albumin:creatinine ratio; BNP, B-type natriuretic peptide; CKD, chronic kidney disease; eGFR, estimated glomerular filtration rate; HCC, hierarchical condition category; NT-proBNP, N-type pro-B-type natriuretic peptide; PCR, urine protein:creatinine ratio

Separately, we used laboratory data from the same 2021 calendar year to identify patients with diabetes (HbA1c ≥ 6.5%, [23] or heart failure (B-type natriuretic peptide > 100 pg/ml, or N-terminal pro-B-type natriuretic peptide > 210 pg/ml, in the latter instance using European Society of Cardiology guidelines for patients older than 50 years; [24, 25]; see Fig. 1). For patients with diabetes, we determined whether guideline-recommended laboratory screening for CKD had been performed in the same 12 months of calendar year 2021: eGFR and urine testing (albumin:creatinine ratio/ACR or protein:creatinine ratio/PCR, [26].

With these inclusion criteria, we sought to identify CKD patients with the comorbid risk factors of diabetes and heart failure, and to also assess guideline-based screening for CKD in patients with diabetes.

### Financial considerations

Beyond the primary objective of identifying the clinical opportunity for unidentified CKD, our second objective was to provide information on the financial impact of not identifying CKD patients. This was by: (a) identifying missed billing opportunities by examining the proportion of CKD patients with a laboratory diagnosis of CKD that was not documented in billing data; and (b) imputing the financial impact of these missed opportunities under the risk adjustment arm of the U.S. value-based payment system.

The CMS model of risk stratification creates an individual’s clinical profile, using the Hierarchies of Chronic Conditions (HCC) to characterize the person’s illness level within each disease process, and the accumulated effects of comorbid disease conditions [27]. Laboratory data informs a significant proportion of HCCs, including CKD, so can significantly improve accuracy of a condition’s diagnosis and hence, its hierarchical position in a patient’s risk-adjusted clinical profile. This has relevance both for the expected clinical management and treatment for that patient, and the related costs of providing such health care.

**Fig. 2:**
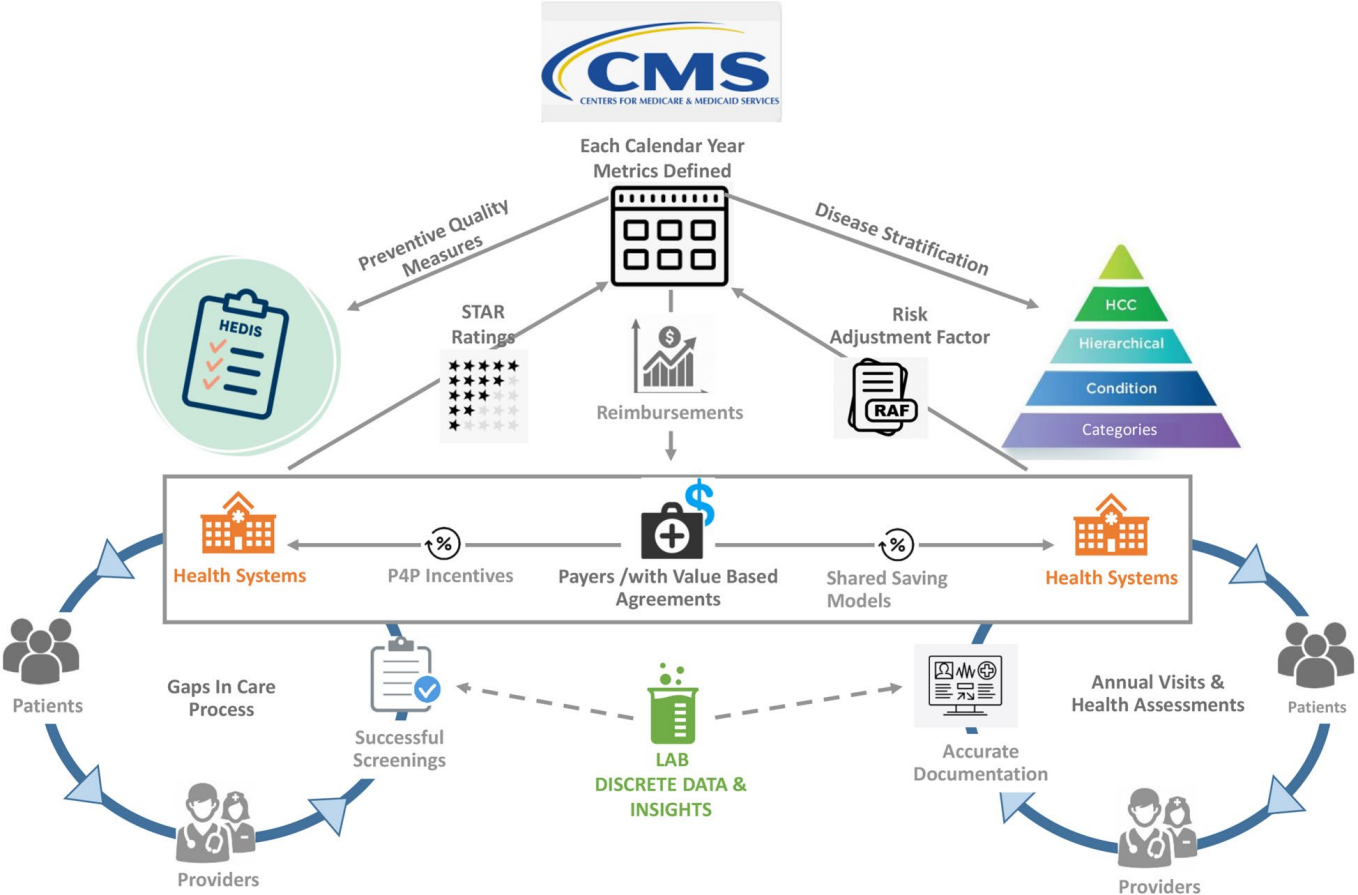
Schematic showing the role of the clinical laboratory in funds flow for value-based payment systems. Under the system managed by the U.S. Centers for Medicare & Medicaid Services (CMS), data from clinical laboratories (bottom center) informs both Pay-for-Performance (P4P, left) and Shared Savings (right) payment systems. Left is a typical Health Effectiveness Data and Information Set (HEDIS) value-based P4P program: first, CMS publishes measures, parameter definitions and thresholds for compliance rates for success (the “STAR” rating system); second, payers develop member reports that identify these cohorts for screenings; third, health systems enter into group insurance coverage agreements with the payer, involving P4P measures; fourth, the health system performs services to successfully deliver health care and close gaps in care delivery; fifth, health system performance is translated into ratings (5 star scale, 5 being best) for STAR outcomes, which then informs CMS reimbursement to the payers. Payers in turn bonus the respective health system for those attributed patients, on the basis of P4P metrics. Right is a typical Share Savings model: first, CMS attributes patients to an entity (payer and/or an Affordable Care Organization/ACO); second, patients start with a default risk score value and need to be assessed annually for their conditions using Heirarchical Condition Categories (HCC); third, patients attend their annual visits and the provider updates their medical record with the relevant ICD-10 codes for existing and potential new conditions (which informs assignment of HCCs); fourth, the Risk Adjustment Factor (RAF) is then used to update each attributed patient's risk score to ensure that the financial costs of their receiving health care are aligned to their risk profile. Although the clinical laboratory currently receives no financial incentives through either set of programs, the clinical information generated by the clinical laboratory is a crucial component of either system being able to operate

**Fig. 3:**
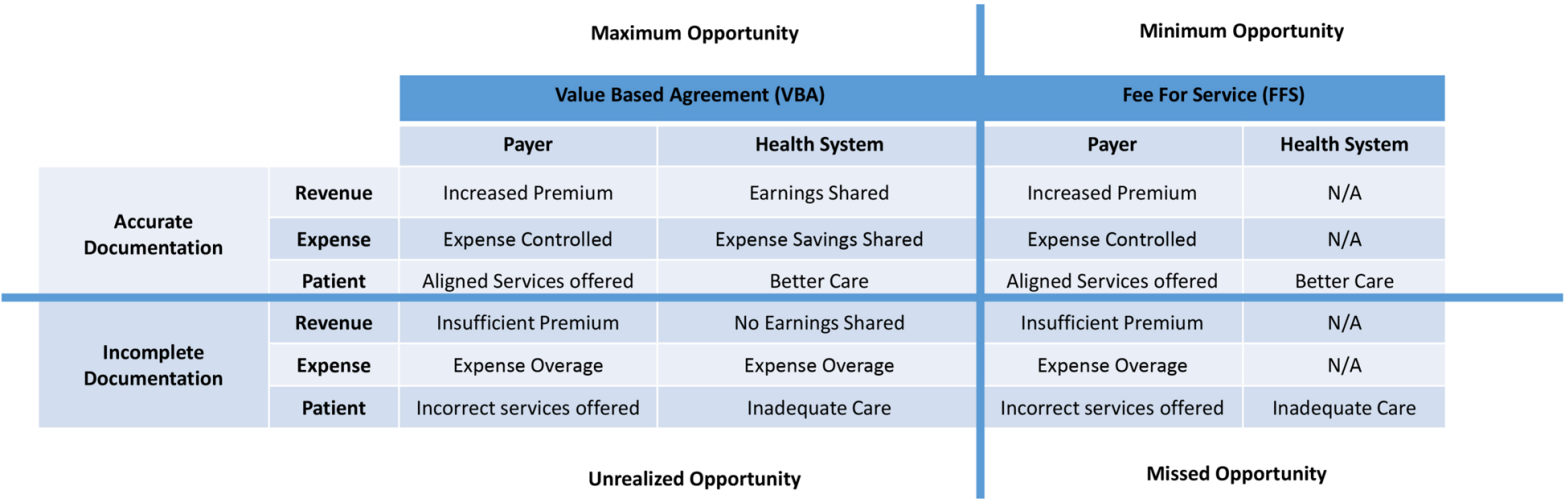
Relationship between value-based versus traditional fee for service approaches to risk adjustable conditions. This matrix depicts the perspectives of pertinent stakeholders (payer, health system, patients) in value-based versus fee for service systems, showing the impact of accurate versus incomplete documentation. Only the value-based system aligns all three stakeholders with documentation and better delivery of health care. The consequences of incomplete documentation of chronic health conditions start with, but are not limited to, inadequate health care for the patient. Not shown are the four tiers of a value-based payment shared savings system: maximum opportunity (where all stakeholders derive benefit with shared incentives); minimum opportunity (where the payer derives benefit, but the patient and health system are excluded from shared payment incentives); missed opportunity (where requisite metrics are not achieved and the stakeholders do not benefit); and unrealized opportunity (where all stakeholders could benefit due to shared incentives, but documentation remains suboptimal)

Figure 2 provides a conceptual framework for placement of the clinical laboratory within the U.S. value-based payment system. The left half depicts the pay-for-performance (P4P) mechanisms for value-based payments, which involve HEDIS quality measures and the CMS Medicare “STAR” Rating Program applied to health systems on the basis of the HEDIS metrics (see Figure legend for definitions of acronyms). The risk-adjusted reimbursement system used by Medicare Advantage is depicted on the right half of the schematic. The schematic intentionally places the “Lab” at the bottom center, given the important actionable information emanating from this source. The structured data elements shown in Fig. 2 show that the clinical laboratory can systematically identify not just patients with each of the three disease conditions (CKD, diabetes, and heart failure), but also can readily risk stratify the patients who have two or even three of those conditions. Figure 3 provides further consideration of the benefits to the payer, health system, and most importantly, patient, when there is accurate documentation of disease conditions, versus when documentation is incomplete.

### Data extraction and analysis

Descriptive statistics for the primary and secondary objectives were compiled and analyzed independently at the three study sites. All data obtained for this study were de-identified, ensuring that the research was conducted in a blinded fashion, protecting patients’ privacy, and eliminating bias in the results. Data were not harmonized across sites, and only aggregate de-identified data were shared under a data use agreement.

At Northwell Health, patient demographics and relevant laboratory results (eGFR, ACR, PCR, HbAlc, BNP and NT-ProBNP,) were extracted from the Laboratory Information System (LIS; Cerner Millennium, North Kansas City, MO, USA). Laboratory test codes were identified using the Current Procedural Terminology (CPT) codes that were agreed upon by the participating institutions, these test codes and associated results were pulled from the LIS system using custom Structured Query Language (SQL) queries. Clinical diagnosis data for CKD, diabetes, and heart failure were acquired using ICD-10 codes from electronic health records. HCC information was extracted from Northwell’s claims application (Clinovations, Optum Insight, Inc., Eden Prairie, MN, USA); only HCCs that were identified as “closed” (through claims data) were included for the comparison. Unique patient identifiers were used to ensure the removal of duplicate patients. To validate the integrity of the data, a subset of patients was selected for manual inspection. This manual verification included cross-referencing the data with primary sources, such as the electronic medical record, to confirm the accuracy of key variables and outcomes. Additional quality measures for the extracted ICD-10 code and HCC files included screening for allowed characters, batch totals, consistency, format, and uniqueness.

At the University of Vermont, the electronic medical record database was queried using EPIC Beaker Reporting Workbench and Slicer Dicer for the data fields described in the protocol. Data were extracted as a.csv file into Microsoft Excel (Microsoft, Redmond, WA, USA). Data were further scrubbed to remove all patients meeting exclusion criteria. Laboratory data were further refined by removing all ACR and Protein-to-Creatinine Ratios that contained a “ < ” or was not able to be calculated due to low urine creatinine level. For each individual patient laboratory test, the ICD-10 code attributed at any time point in the study period was linked to that laboratory test result to aid in measurement of secondary objective. The patient’s medical record number was used to ensure that for all descriptive statistics and study objectives, only a single unique patient was counted, by removing any duplicates once laboratory-based CKD, heart failure, or diabetes was identified. Data were validated using the manual methods of allowed characters, batch totals, consistency, format, and uniqueness.

For the Geisinger Health System, the data for this study were extracted by analysts from the Geisinger Health Data Analytics team. Additionally, a data sample exchange form was used to determine the need for data usage agreements between the Geisinger Health Data Analytics organization and external institutions with whom data sharing was required. Two hundred random charts were reviewed to ensure the data represented the results expected from the query and was coded appropriately in light of clinical and laboratory findings in the patient chart prior to submitting the final deidentified data set.

For the second objective, the authors identified the financial impact of missed risk adjustment payments for Stage 3 and Stage 4 CKD from the study population identified with laboratory results and no corresponding ICD-10 or HCC codes for CKD. From this sub-group, the number of patients enrolled in Medicare Advantage (MA) and Affordable Care Act (ACA) plans that use CMS value-based risk adjustment payments were estimated from state reported data for each participating site. Risk adjustment payments in 2021 used a blended model from prior years based on demographics, socioeconomic factors such as location, and disease burden. The blended payment model or Risk Adjustment Factor (RAF) scores apply to MA and ACA patients for federal government reimbursement. While study institutions may have had reimbursement for improved HEDIS measures and STAR ratings or additional contractual arrangements for reimbursing Stage 3 and Stage 4 CKD patients not enrolled in MA or ACA plans, this information was not available to the authors. As a result, only estimated MA and ACA populations were used for the missed risk adjustment payment analysis. This may have resulted in a conservative number of CKD patients with missed risk adjustment reimbursement. All estimates used publicly available 2021 CMS enrollment and RAF data, to align with the time frame for the study data collected.

## Results

**Fig. 4:**
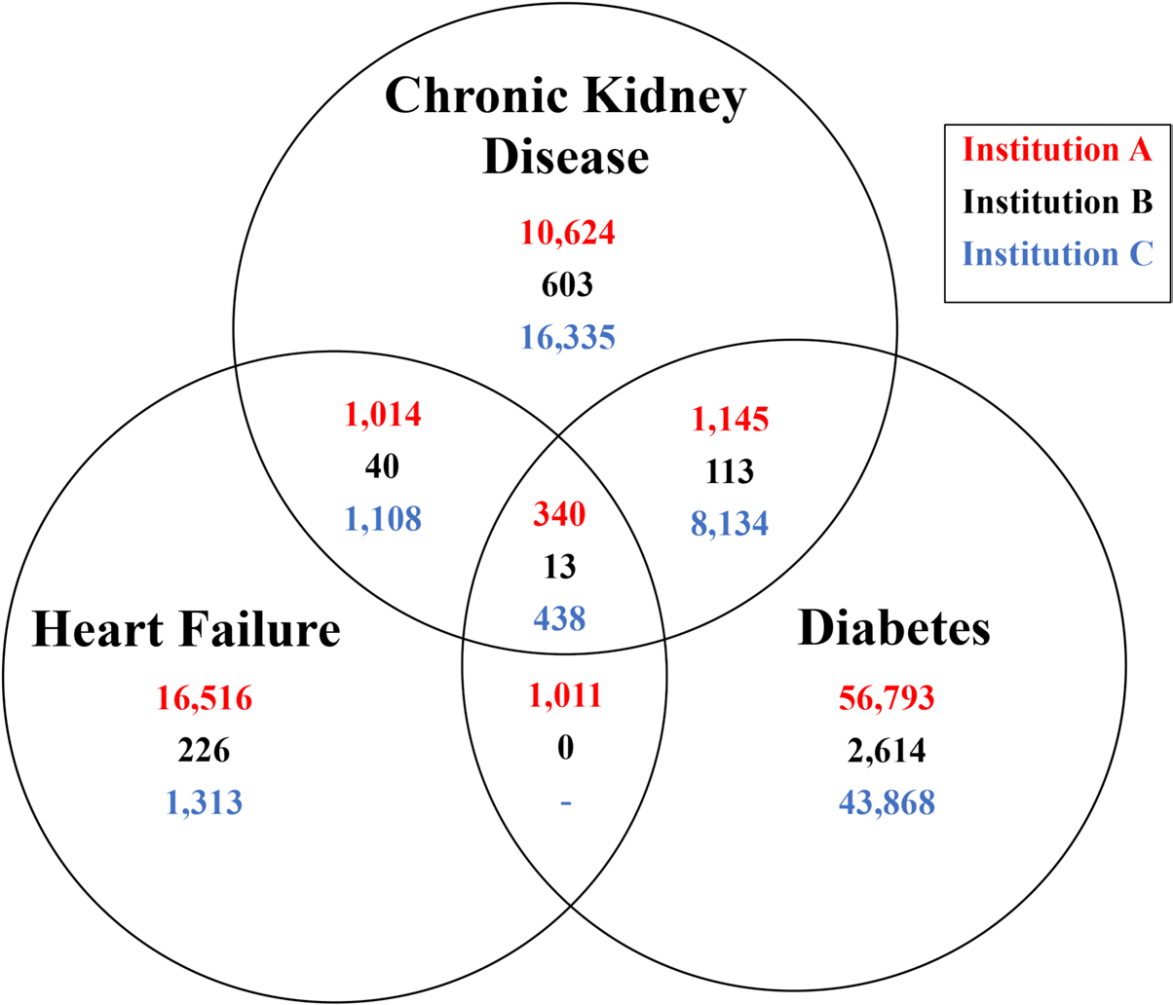
Venn diagram of the study population (number of persons), identified by laboratory data. Laboratory criteria for chronic kidney disease, diabetes, and heart failure are given in Fig. 1. For institution C, data were not available for coexistence of heart failure and diabetes in the absence of CKD. CKD, chronic kidney disease; DM, diabetes mellitus; HF, heart failure

In the three participating institutions, patients with laboratory evidence of and/or ICD-10 or HCC coding for chronic kidney disease, diabetes, and heart failure were identified using the criteria in Fig. 1, including simultaneous occurrence of CKD with either-or-both diabetes or heart failure. The number of patients identified at each institution are given in Fig. 4. The differences between the three institutions in absolute numbers of patients identified reflects the size of their respective patient populations. The frequency of diabetes accompanying CKD was 1485 (11.3%), 126 (16.7%), and 8572 (33.0%) from Institutions A, B and C, respectively. For CKD accompanied with heart failure, 1354 (10.3%), 153 (20.2%), and 1546 (5.9%) of CKD patients from institutions A, B, and C, respectively, were identified with the two conditions.

Conversely, of those patients with diabetes, the institutional variation in identification of CKD was even greater: Institution A identification of CKD among patients with diabetes was 1485 of 59,289 (2.5%); Institution B was 126 of 2740 (4.6%) and Institution C was 8572 of 52,440 (16.4%). Of patients with heart failure, institutional identification of CKD was: for Institution A, 1354 of 18,881 (7.2%); Institution B was 53 of 279 (19.0%) and Institution C was 1546 of 2859 (54.1%). Identification of patients with diabetes and with heart failure for the coexistence of CKD at institution C thus exceeded that of institution A by approximately sevenfold (16.4% vs. 2.5%; 54.1% vs. 7.2%, respectively). Although these data point to widely variable practices in the identification of these comorbid conditions, they do not permit assessment of whether our results reflect underlying differences in the prevalence of these conditions in our respective populations, or only differences in laboratory testing and coding practices.

The number of patients with diabetes on the basis of a laboratory finding of HbA1c ≥ 6.5% in calendar year 2021, but without evidence of guideline-based laboratory screening for CKD during the same 12 months, is shown in Table 1. The percentage of patients without CKD screening ranged from 59 to 83% at the three institutions.

**Table 1:** Screening for CKD in persons with diabetes. Persons at each institution with diabetes based on laboratory criteria (HbA1c ≥ 6.5%, laboratory test performed between January 1, 2021 to December 31, 2021), but without guideline-based CKD screening during the same 12 months (both an eGFR and either a urine albumin:creatinine ratio or protein:creatinine ratio)

	**Institution A**	**Institution B**	**Institution C**
Persons with diabetes (HbA1c ≥ 6.5%) with *no screening for CKD* *during the same 12 months*	**59%** (34,384/58,278)	**83%** (2,274/2,740)	**77%** 40,378/52,440)

Looking specifically at in-system laboratory testing versus administrative coding, there was substantial variation between the three institutions in the identification and documentation of patients with CKD (Table 2). The percentage of patients who had laboratory results only for CKD and no corresponding ICD-10 or HCC coding (Table 2, row 1) ranged from 32% (Institution B) to 78% (Institution A). Institution A had the smallest percentage of patients (4%) with both laboratory results of CKD and evidence of a diagnostic code for CKD being included in billing information for calendar year 2021; while Institution C had the greatest percentage (22% (;Table 2, row 2). When viewed from the perspective of diagnosis coding on the basis of only in-system laboratory test results (Table 2, row 3), Institution A did so for only 5% of its patients, in comparison with 37% and 25% for Institutions B and C, respectively.

**Table 2:** Identification of Chronic Kidney Disease. First, second and fourth rows: Percent of study population at each institution identified with CKD based on laboratory results alone, laboratory results plus documented ICD-10 or HCC diagnosis code, and documented ICD-10 or HCC diagnosis code and no laboratory results, respectively. Absolute numbers of patients in each category are shown in the parentheses as the numerator; total number of patients with CKD by either-or-both laboratory and/or diagnosis code are given as the denominator. Third row: Percent of the subset patients at each institution who had in-system laboratory evidence of CKD and had a diagnosis code for CKD; absolute numbers are shown in the parentheses as numerator (from second row), total number of patients with evidence of CKD on the basis of in-system laboratory data are shown as the denominator (sum of first and second rows).

	**Institution A**	**Institution B**	**Institution C**
In-system Lab Results Only	**78%** (12,478/16,063)	**32%** (487/1511)	**66%** (19,433/29,277)
In-system Lab plus Diagnosis Code	**4%** (645/16,063)	**19%** (282/1511)	**22%** (6,582/29,277)
Diagnosis code for in-system Lab Results	**5%** (645/13,123)	**37%** (282/769)	**25%** (6,582/26,015)
Diagnosis Code Only	**18%** (2,940/16,063)	**49%** (742/1511)	**11%** (3,262/29,277)

The overall percentage of patients with only ICD-10 codes but no supporting in-system laboratory values for CKD (Table 2, row 4) ranged from 11% (Institution C) to 49% (Institution B). A potential explanation for the disproportionately high percentage of ambulatory patients cared for at Institution B that had diagnostic coding for CKD but no accompanying laboratory test data may have been the referral pattern of their network. Specifically, patients identified with CKD outside their health system may have been receiving ancillary care, without repeat laboratory testing being performed at Institution B. For institutions A and C, respectively, the 18% and 11% of patients with diagnosis codes only may also be a reflection of referral patterns, and/or performance of laboratory testing by outside laboratories that were recognized as in-network providers under the patients’ insurance plans.

 In turn, for those patients identified by in-system laboratory data as having CKD, but without accompanying ICD-10 or HCC documentation in the electronic medical record, their subcategorization into CKD Stages 3A, 3B and 4 are given in Table 3. The highest percentage of such patients was in CKD Stage 3A, and was consistent across all three institutions (range 58% to 71%).

**Table 3:** Stage of Chronic Kidney Disease. Percent of study population at each institution identified with CKD stages 3A, 3B, and 4 based on laboratory results alone, without a documented ICD-10 diagnosis or HCC code (see Table 2, row 1). Stage 3A, eGFR between 45 to 59 ml/min/1.73m2 for 3 months or more; Stage 3B, eGFR between 30 to 45 ml/min/1.73m2 for 3 months or more; Stage 4, eGFR between 15 to 29 ml/min/1.73m2 for 3 months or more. For each institution, absolute numbers of patients in each category are shown in the numerator in each parentheses; the total number of patients with CKD Stage 3 or Stage 4 by eGFR but with no corresponding ICD-10 or HCC code in the electronic medical record is shown as the denominator)

CKD Stage by eGFR but with no ICD-10 or HCC code	Institution A	Institution B	Institution C
3A	61% (7,612/12,478)	58% (282/487)	71% (13,797/19,433)
3B	26% (3,244/12,478)	31% (151/487)	22% (4,081/19,433)
4	13% (1,622/12,478)	11% (54/487)	7% (1,555/19,433)

For the second objective, the authors estimated the financial impact of RAF scores based on Medicare Advantage Capitation Rates and Medicare Advantage and Part D Payment Policies and Risk Adjustment Model coefficients, as announced by CMS (see Supplement). Under this system, provider entities receive annual specified reimbursements from Medicare for individuals enrolled in these programs, in accordance with the RAF scores for comorbidities documented as HCCs for each individual. Table 3 reports a total of 29,167 patients at the 3 institutions with laboratory-identified only CKD Stage 3, and 3231 patients with laboratory-identified only CKD Stage 4. In Table 4, we estimated on a per-state basis that 9.9% of those patients would be beneficiaries enrolled in Medicare Advantage or ACA Marketplace programs (see Supplement for details). For each Medicare Advantage patient with undocumented CKD Stage 3 or Stage 4, in 2021 the payer responsible for the benefits plan would not have received an annual Medicare Advantage reimbursement of $581 or $1,987, respectively. The total financial impact of such unrealized Medicare Advantage reimbursement is given in the final column; an imputed total of $2,285,090 annually.

**Table 4:** Financial impact of undocumented Chronic Kidney Disease. Estimated unrealized reimbursement opportunity for undocumented CKD for Medicare Advantage alone, for the 3 institutions combined in calendar year 2021

Annual Reimbursement per Beneficiary	Total Individuals with CKD documented by Lab only^a^	Estimated number of Beneficiaries^b^	Annual Reimbursement per Beneficiary (2021)^c^	Unrealized Risk Adjustment Reimbursement^d^
Medicare Advantage CKD Stage 3	**29,167**	**2866**	**$ 581**	**$1,665,146**
Medicare Advantage CKD Stage 4	**3231**	**312**	**$1,987**	**$619,944**
ACA Marketplace CKD Stage 4	**3231**	**68**	**$8,377**	**$569,636**

*ACA* Affordable Care Act, *CKD* chronic kidney disease

^a^From Table 3. ^b^Estimated number of Medicare Advantage beneficiaries on the basis of % Medicare beneficiaries by state in 2021 (https://www.kff.org/medicare/state-indicator/total-medicare-beneficiaries/?currentTimeframe=0&sortModel=%7B%22colId%22:%22Location%22,%22sort%22:%22asc%22%7D; accessed February 11, 2024), pro-rated to % of Medicare beneficiaries enrolled in Medicare Advantage in 2021 (https://www.kff.org/medicare/issue-brief/a-snapshot-of-sources-of-coverage-among-medicare-beneficiaries/#:~:text=In%202021%2C%20Medicare%20Advantage%20covered,%25%20of%20all%20eligible%20beneficiaries; accessed February 11, 2024); estimated Affordable Care Act (ACA) Marketplace beneficiaries estimated on the basis of % of population enrolled (https://www.kff.org/affordable-care-act/state-indicator/total-marketplace-enrollment/?currentTimeframe=0&sortModel=%7B%22colId%22:%22Location%22,%22sort%22:%22asc%22%7D; accessed February 13, 2024). ^c^Annual Medicare Advantage Reimbursement per Beneficiary in 2021 based on Medicare Advantage payment policies (https://www.cms.gov/files/document/2021-announcement.pdf; https://www.cms.gov/CCIIO/Resources/Regulations-and-Guidance/Downloads/Final-2021-Benefit-Year-Final-HHS-Risk-Adjustment-Model-Coefficients.pdf, both accessed February 11, 2024); Affordable Care Act (ACA) Marketplace Reimbursement per Beneficiary in 2021 based on Final Risk Adjustment Model Coefficients (https://www.cms.gov/CCIIO/Resources/Regulations-and-Guidance/Downloads/Final-2021-Benefit-Year-Final-HHS-Risk-Adjustment-Model-Coefficients.pdf; accessed February 13, 2024). ^d^Multiplicand of Estimated number of Beneficiaries and Annual Reimbursement per Beneficiary (2021). See Supplement for further details

Table 4 also shows the imputed enrollment of patients in Affordable Care Act (ACA) Marketplace products, based on % enrollment per state. This latter calculation is shown only for CKD Stage 4, since in 2021 the ACA Marketplace did not provide reimbursement for CKD Stage 3. Although the number of imputed beneficiaries in the ACA Marketplace is low (n = 68), the unrealized Risk Adjustment reimbursement is still substantial ($569,636), owing to the much higher annual Risk Adjustment per beneficiary. Although the risk adjustment-based reimbursement would have applied only to about 1 in 10 patients with undocumented CKD Stage 3 or 4, these calculations give an estimate of the financial impact of failure to document CKD on an annual basis. These calculations do not include the potential increases in RAF values for patients with coexistent diabetes or heart failure (in the presence of CKD, 6), so may not reflect the total financial impact of under-documentation of CKD.

## Discussion

Chronic diseases such as kidney disease, hypertension, and diabetes are asymptomatic in the early stages and can benefit from early detection by laboratory testing. Early detection in turn enables less costly interventions and improved quality of life. In this multi-institutional study, we demonstrated in both Tables 2 and 3 varying but substantial opportunities for the clinical laboratory to identify patients whose electronic medical record did not document the presence of CKD during the calendar year under study. The published literature supports the premise that failure to document CKD through coding serves as a surrogate marker for physicians failing to incorporate this knowledge into patient management [28]. Hence, our findings underscore the importance of using data streams generated from current clinical workflow, to ensure early identification of patients with laboratory markers diagnostic of CKD.

Our findings in Table 1 document that a substantial fraction of patients with laboratory evidence of diabetes were not being tested for the presence of CKD using the “Kidney Health Evaluation for Patients with Diabetes” measure [16]. Such testing is in fulfilment of HEDIS quality measures for screening of patients with diabetes for eGFR and urine ACR or PCR within that calendar year [16]. These data are in keeping with previously documented low rates of adherence to CKD assessment in patients with diabetes or hypertension [29, 30]. Moreover, only half of patients meeting criteria for nephrologist referral are under the care of a nephrologist [31].

Conversely, our findings in Table 2 corroborate published reports in which a substantial portion of patients with laboratory evidence of CKD lack a corresponding diagnosis code for CKD [29, 32–35]. A review of 2011 beneficiaries enrolled in Medicare with laboratory data indicating CKD found that only 11.8% had evidence of clinical recognition through diagnosis codes [36]. Reviewing 2014 data, CMS reported that over 70% of Medicare beneficiaries whose laboratory tests indicated CKD were not diagnosed with CKD [37]. The fact that the 2014 CMS data were similar to our findings for CKD under-documentation in 2021 underscores the continued challenge of closing this health care gap [38].

From the standpoint of who is responsible for documentation of disease conditions, for hospitalized patients in the U.S., the administrative coding for diagnoses upon discharge is performed by professional coders [39]. However, in the ambulatory setting, the providing physician holds the substantive responsibility for coding of patient encounters, part of administrative tasks that constitute almost half of their time daily [39]. The low rates of coding documentation may be strongly influenced by the burden placed on primary care physicians for performing these administrative tasks. Indeed, the potential for AI-assisted identification and coding of patients with CKD is currently being explored for both the primary care and subspecialty nephrology settings [40, 41].

In Table 4 and as explained in the Supplement, we estimated the risk-adjusted reimbursement not received for clinical care of patients at our three institutions. This estimate was only for the number of patients imputed to be in Medicare Advantage and ACA Marketplace risk-adjusted payment programs, with CKD Stage 3 or 4 and no corresponding ICD-10 or HCC coding. We calculate for 3246 patients that a total of $2.85 M was not realized in risk-adjusted reimbursement. Considering that there are about 37 million adults in the U.S. with CKD, most of whom are undiagnosed [1, 2], our financial estimate for unrealized reimbursement for about 1% of the U.S. population with CKD is a sobering reminder of the hidden costs of this disease. As the U.S. fee-for-service reimbursement system is phasing out in favor of a value-based payment system, including for care of patients with CKD [42–44], the financial jeopardy for health care providers who fail to document and provide pro-active care for patients with chronic conditions will only increase [45].

The ability to realize the financial opportunity in Table 4 depends on the type of agreement a health system has with a payer or with CMS, particularly if participating in Value-Based P4P programs (the left side of Fig. 2) or through engagement in Shared Savings risk-adjusted value-based agreements (the right side of Fig. 2, upon which the calculations in Table 4 are based). Under either form of agreements, the clinical laboratory contribution to a coordinated effort can support better-managed care for the patient, and alignment with the financial incentives that are part of these programs. An understanding of U.S. healthcare payment models is thus relevant to the emerging role of clinical laboratories in providing additional value beyond diagnostics.

Taking our Tables 1– 4 collectively, non-identified patients with CKD are not appropriately risk-adjusted for this chronic condition, and will then have poorer-than-expected quality outcomes and consumption of health care resources. The worse-than-expected clinical and cost outcomes either degrade the HEDIS ratings under a value-based Pay-for-Performance paradigm, and/or erode potential Shared Savings under value-based risk-adjusted payment systems. Both of these outcomes have negative financial impact on the payer and, in turn, the health system providing care [18].

Evidence is emerging that value-based payment systems are effective. A comparative analysis of spending patterns between Medicare Advantage and the Medicare Shared Savings Program revealed that spending within the latter was 30% higher than in Medicare Advantage [46]. This finding assumes added importance when considering projections that the Medicare Advantage program, which has grown from 19% in 2007 to 51% in 2023, is anticipated to encompass over 60% of Medicare beneficiaries by 2030 [47]. Consequently, health systems must not only comprehend risk adjustments but also excel in identifying and effectively managing patients with conditions that affect risk adjustment.

This study had limitations that could impact the clinical and financial outcomes reported. The CKD stage and concomitant chronic disease identification could be impacted if the patient’s laboratory results were not available in the electronic medical record of a given institution. This may have been particularly true for institution B, for which a large referral network may have led to the laboratory testing being performed outside the health system, and referral patients with only ICD-10-coded CKD being cared for by the health system. Regardless, for all three institutions the same concern of under-documentation must be raised for laboratory testing that was performed by outside laboratories. Specifically, if the low rates of identifying CKD from in-system laboratory data documented in Table 2 apply to laboratory testing performed by outside laboratories, the number of patients receiving health care from our systems but not identified as having CKD could be higher than reported herein.

Second, our analysis of administrative coding data for CKD does not take into account the widely varying sensitivity and specificity of coding practices [48, 49]. Beyond the fact that the transition of the U.S. coding system in 2015 from ICD-9 to ICD-10 has not improved identification of CKD stages 3 and 4 [50], the ICD-10 system continues to identify only the primary stages of CKD, without distinguishing between stage 3A and 3B. Moreover, the sensitivity and specificity of serum-creatinine based estimation of glomerular filtration rate is dependent on patient characteristics, particularly lean muscle mass and physical activity but also age, gender, ethnicity and dietary protein intake [51, 52]. Rapidly changing patient physiologic status, drug-induced inhibition of renal tubular secretion, and interfering substances also affect serum creatinine levels [53]. The sensitivity of serum creatinine may thus be poor, especially in the elderly [54]. Serum cystatin C-based eGFR is not influenced by the above confounding factors [55, 56], and is thus advocated as being superior to serum and urinary creatinine as a marker of kidney function [57]. Nevertheless, serum creatinine-based eGFR is still considered to be the standard method for assessment of renal function as it is routinely measured in real-world clinical practice [53], with serum cystatin C-based eGFR or a combined creatinine-cystatin C equation serving as an additional test for confirmation [58, 59]. In turn, B-type natriuretic peptide (BNP) and N-terminal pro-brain natriuretic peptide (NT-pro-BNP) are the commonly used biomarkers for predicting heart failure and left ventricular dysfunction [60]. However, BNP may not increase proportionally in heart failure patients, and NT-pro-BNP is primarily excreted by the kidney, so plasma concentrations of this latter biomarker may be intrinsically higher in patients with CKD [61, 62]. Hence, these biomarkers may have at best moderate specificity for heart disease. These many considerations about laboratory testing underscore the need for close attention to the serum creatinine-based eGFR test data that does enter the electronic health record, as it is but a first step in evaluating patients for kidney disease and as a fundamental risk factor for cardiovascular disease [58]. Argument is also made that it is the serial evaluation of the creatinine-based eGFR that is an important indicator of CKD, as it is identification of the eGFR “rapid progressors” that improves sensitivity and specificity of this laboratory screening test [63].

Third, obtaining measures of kidney function in an observational study may be subject to ascertainment bias, based on test ordering patterns as part of the clinical workflow [40]. Fourth, at the institutions in the study, we did not specifically identify the number of patients enrolled in value-based contracts, i.e., that provide for risk adjustment reimbursement. The latter two limitations may have led to an underestimation of the actual number of patients that could be identified and the financial impact or finding these patients. The authors initially attempted to collect information on the type of insurance for each patient identified, but not all sites had this information available. As a result, the number of Medicare Advantage and ACO Marketplace patients were instead imputed for each institution based on publicly available state data for beneficiary enrollment in value based payment systems. Fifth, this was a retrospective study, so does not provide information on how programmatic initiatives by these institutions may or may not have been making progress on improving identification and clinical management of patients with CKD. Finally, it is important to note that risk adjustment requires the patient to receive a specifically qualified physician visit within the year an adjustment is requested, which would be reflected in billing data for Current Procedural Terminology (CPT) coded health care encounters. This study did not review the occurrence, nor absence, of CPT coding for study patients in the year for which the data were extracted.

Lastly, an important next step in our work will be to examine the longitudinal outcomes of patients we identified in this study, to assess whether identification of CKD in their electronic health record was associated with better outcomes than for patients with laboratory evidence of CKD but who were not identified as such in the record.

## Conclusion

This observational study demonstrates how clinical laboratories can provide value beyond diagnostics using longitudinal data for the identification of chronic kidney disease, stratify subgroups of patients to identify risk, identify gaps in clinical care associated with quality measures such as HEDIS, and capture missed reimbursement through risk adjustment factors not documented in the billing system. The information generated by clinical laboratories constitutes a major opportunity for program design and implementation, both to improve the clinical outcomes of this population of patients, and to deliver health care in a more cost-effective fashion. This last concept is the founding premise of Clinical Lab 2.0 [21]. The current study underscores the great need for advancing this mission [64]. It is our fervent hope that future studies will provide valuable information for how to enhance the care of patients with this chronic condition.

## Supplementary Information

Supplementary Material 1.

## Data Availability

Data is provided within the manuscript.

## Abbreviations

ACA: Affordable Care Act
ACR: Urine albumin:creatinine ratio
BNP: B-type natriuretic peptide
CKD: Chronic kidney disease
CMS: Centers for Medicaid & Medicare Services
CPT: Current Procedural Terminology
DM: Diabetes mellitus
eGFR: Estimated Glomerular Filtration Rate
HCC: Hierarchical condition categories
HEDIS: Healthcare Effectiveness Data and Information Set
HF: Heart failure
IRB: Institutional review board
KDIGO: Kidney Disease: Improving Global Outcomes
MA: Medicare Advantage
NCQA: National Committee for Quality Assurance
NKF: National Kidney Foundation
NT-ProBNP: N-terminal pro-B-type natriuretic peptide
PCR: Urine protein:creatinine ratio
PSFF: Project Santa Fe Foundation
RAF: Risk adjustment factor
