# Supplementary material for: A retrospective multi-site examination of chronic kidney disease using longitudinal laboratory results and metadata to identify clinical and financial risk
Source: BMC Nephrol. 2024 Dec 6;25:447. doi: 10.1186/s12882-024-03869-4 (PMC11622455; doi:10.1186/s12882-024-03869-4)
Supplement: Supplementary file 1 — Supplementary Material 1. [file 12882_2024_3869_MOESM1_ESM.docx]

**SUPPLEMENT**

**A Retrospective Multi-site Examination of Chronic Kidney Disease Using Longitudinal Laboratory Results and Metadata to Identify Clinical and Financial Risk**

Fung M, Haghamad A, Montgomery E, Swanson K, Nowak SA, Wilkerson ML, Wilburn C, Kavus H, Swid MA, Okoye N, Ziemba YC, Ramrattan G, Stathakos K, Macy J, McConnell J, Lewis MJ, VanNess R, Crawford JM

**Calculation of Risk Adjustment Factors for Heirarchical Condition Categories**

**Introduction**

Under the United States value-based payment system for health care delivery, there are several types of risk adjustment models, but two of the major models that use the Hierarchical Condition Category (HCC) system are^[[1]](#footnote-1)^:

1. CMS-HCC risk adjustment model for Medicare/Medicare Advantage, which calculates risk payments for the following year
2. HSS-HCC risk adjustment model for commercial payers including the Affordable Care Act (ACA) Marketplace exchanges created through Obamacare which calculates risk for the current year

The Medicare Advantage insurer takes the financial risk for each beneficiary, calculated from documentation of the beneficiary’s HCCs from the prior year. In so doing, the Medicare Advan­tage insurer assumes the financial responsibility of paying for the covered healthcare expenses of that specific beneficiary for the current calendar year. The insurer receives a monthly base prem­ium from Medicare Advantage, to which is added a monthly HCC risk-adjusted payment calcu­lated from the prior year’s documentation of HCCs. The ACA Marketplace exchange works much like Medicare Advantage, but due to a younger population, the monthly risk adjustment payment is less and the payments are applied to the current year.

Both models use a risk adjustment factor (RAF) score to estimate expected future health costs for each beneficiary. The RAF score is specifically calculated for each beneficiary using demo­graph­­ics, socioeconomic factors such as location, and disease burden. The monthly risk adjustment payment is adjusted (upward or downward) using these factors:

1. Demographics such as age/sex and location (socioeconomic factors). CMS publishes this rate which ranges from $800-1200 per patient per month, with urban areas having a higher amount. Every 1-2 years, the Centers for Medicare and Medicaid Services (CMS) releases a table of population demographics across the U.S.. The numbers are based on estimated cost data as well as deliberate policy choices. The “conversion factor” for demographics usually range from 0.6-1.2.
2. Disease burden of the beneficiary is based on HCC documentation with ICD10 codes and annual documented patient visits to a health care provider. The risk adjustment model coefficients for HCCs is published annually and the methodology for calculating these payments is revised almost annually. This means that the reimbursement for these patients can changes annually.
3. In 2021, a blended model for risk adjustment calculations was used that included 75% of the 2020 CMS-HCC model and 25% of the 2017 CMS-HCC model to incorporate more inpatient and encounter data^[[2]](#footnote-2)^.

Chronic kidney disease (CKD) stage 3 (HCC N18.30) has clinically significant ramifica­tions for comorbid disease management and medication usage. Treating and managing a patient with CKD stage 3 before progression to later stages has a significant impact on the health of the patient. CMS has not always recognized CKD stage 3 as a reimbursable risk adjustment condition.

The fundamental premise is that bene­fic­i­aries with documented HCCs from the prior year will incur more costs for their health care in the following year. The rationale for CMS to conduct Risk Adjustment Factors is to pay insurance plans for the risk of the beneficiaries they enroll, instead of calculating an average amount of expenses for Medicare/Medicare Advantage beneficiaries.

**Calculation of Risk Adjustment Factors for Chronic Kidney Disease**

*Study Assumptions for Medicare Advantage:*

1. 2021 information for Medicare Advantage beneficiary calculation^[[3]](#footnote-3)^:
   1. 0.85 as the general age/sex/socioeconomic risk adjustment factor
   2. $1000 per patient per month for the Medicare Advantage reimbursement which is an estimate based on the rate tables
   3. Mean risk adjustment coefficient for CKD stage 3 = 0.0484. Range 0.017- 0.092.
   4. Mean risk adjustment coefficient for CKD stage 4 = 0.187

Range .039-.289.

1. CMS did not recognize CKD stage 3 (eGFR typically ranging 30-59) as a HCC code until the introduction of CMS-HCC version 24 which took effect in 2021. Prior to CMS-HCC version 24, stage 3 CKD was not recognized as part of the HCCs^[[4]](#footnote-4)^.
2. 2021 information for the commercial/ACA market beneficiaries^[[5]](#footnote-5)^:
   1. 0.342 as the general age/sex/socioeconomic risk adjustment factor
   2. $750 per patient per month payment for commercial/ACA market
   3. Mean risk adjustment coefficient of 0.9308 for CKD stage 4. Range 0.898 to 0.990.
3. HSS-HCCs did not recognize CKD stage 3 in 2021.

*Data and References for Medicare Advantage beneficiary 2021 RAF scores*^2,^^[[6]](#footnote-6)^:

1. HCC138 Chronic Kidney Disease, Moderate (Stage 3): 0.069, 0.021, 0.017, 0.043, 0.092
2. HCC137 Chronic Kidney Disease, Severe (Stage 4): 0.289, 0.105, 0.260, 0.138, 0.280, 0.039, 0.201

*How the calculation works for Medicare Advantage beneficiaries using 2021 RAF scores:*

*Demographics + RAF x Payment = Total Payment for CKD Stage 3*

1. 0.85 (average for Medicare) + 0.0484 (mean for CKD Stage 3 based on the 2021 information published by CMS) = 0.8984
2. 0.8984 *X* $1000 per beneficiary per month (using the average, since this amount is driven from socioeconomic factors that we do not have the exact data for) = $898.4 *X* 12 months = $10,781 annual payment with CKD stage 3.
3. The annual payment without documentation of CKD stage 3 = 0.85 *X* $1000 X12 = $10,200.

The unrealized reimbursement for no HCC coding of CKD Stage 3 in Medicare Advan­tage patients is thus $10,781 - $10,200 = $581 for each Medicare beneficiary in 2021.

*Demographics + RAF x Payment = Total Payment for CKD Stage 4*

1. 0.85 (average for Medicare) + 0.187 (mean for CKD 4 based on the 2021 information published by CMS) = 1.037
2. 1.037 *X* $1000 per beneficiary per month (we are using the average since this amount is driven from socioeconomic factors that we do not have the exact data for) = $1037 *X* 12 months = $12,187 annual payment with CKD stage 4.
3. The annual payment without documentation of CKD stage 4 = 0.85 *X* $1000 X12 = $10,200.

The unrealized reimbursement for no HCC coding of CKD stage 4 in Medicare Advan­tage patients is thus $12,187 - $10,200 = $1,987 for each Medicare beneficiary in 2021.

*Study Assumptions for the commercial ACA Marketplace:*

1. 2021 information for commercial/ACA beneficiary calculation^[[7]](#footnote-7)^:
   1. 0.342 as the general age/sex/socioeconomic risk adjustment factor
   2. $750 per patient per month for the commercial/ACA reimbursement which is an estimate based on the rate tables
   3. Mean risk adjustment coefficient for CKD stage 4 = 0.9308 (range 0.0898-0.9900)
2. CKD stage 3 did not have a risk adjustment model coefficient for the commercial/ACA market in 2021. In other words, there were no additional risk payments for CKD stage 3 patients.

*Data and References for ACA Marketplace beneficiaries using 2021 RAF scores*^6^*:*

HCC188 Chronic Kidney Disease, Severe (Stage 4) 0.990, 0.934, 0.898, 0.916, 0.916

*How the calculation works for ACA Marketplace beneficiary using 2021 RAF scores:*

*Demographics + RAF x Payment = Total Payment*

1. 0.342 (average for ACA market with range of 0.167 to 0.517) + 0.9308 (mean of 0.908 with a range of 0.898-0.990 for CKD 4) = 1.2728
2. 1.2728 X $750 per beneficiary per month (average) = $954.60 X 12 months = $11,455 annual payment with CKD stage 4.
3. Annual payment without documentation of CKD stage 4 = .342 X $750 X12 = $3,078

The unrealized reimbursement for no HCC coding in CKD stage 4 = $11,455 - $3,078 = $8,377 for each ACA beneficiary.

**Conclusion**

These calculations demonstrate CMS has recognized the need to identify patients with CKD Stage 3 and Stage 4, and to incorporate financial risk adjustment for the payers incurring the costs of health care received by these beneficiaries.

1. <https://www.cms.gov/files/document/2024-advance-notice.pdf> (Accessed March 5, 2024); <https://www.linkedin.com/pulse/bringing-your-raf-scores-focus-tom-davis-md-faafp/?trk=pulse-article_more-articles_related-content-card> (Accessed March 5, 2024); [BMA_RiskAdjustment_WhitePaper_2018_02_27_v2a.pdf](file:///C:\Users\kathl\OneDrive\Desktop\Project%20Santa%20Fe\CKD\Documents%20on%20HCC%20scores\BMA_RiskAdjustment_WhitePaper_2018_02_27_v2a.pdf) (Accessed March 5, 2024). [↑](#footnote-ref-1)
2. <https://www.cms.gov/newsroom/fact-sheets/2021-medicare-advantage-advance-notice-part-i-risk-adjustment> . Accessed March 6, 2024. [↑](#footnote-ref-2)
3. [Announcement of Calendar Year (CY) 2020 Medicare Advantage Capitation Rates and Medicare Advantage and Part D Payment Policies and Final Call Letter (cms.gov)](https://www.cms.gov/Medicare/Health-Plans/MedicareAdvtgSpecRateStats/Downloads/Announcement2020.pdf) (Accessed March 5, 2024). [↑](#footnote-ref-3)
4. <https://www.aapc.com/discuss/threads/ckd-n18-30-n18-31-n18-32-are-no-longer-hccs-for-2021.176473/> (Accessed March 5, 2024). [↑](#footnote-ref-4)
5. [Final 2021 BY HHS Risk Adjustment Model Coefficients 051120 (cms.gov)](https://www.cms.gov/CCIIO/Resources/Regulations-and-Guidance/Downloads/Final-2021-Benefit-Year-Final-HHS-Risk-Adjustment-Model-Coefficients.pdf) (Accessed March 5, 2024). [↑](#footnote-ref-5)
6. [Announcement2021.pdf](file:///C:\Users\kathl\AppData\Local\Microsoft\Windows\INetCache\Content.Outlook\2NZXBKCP\Announcement2021.pdf)

   [Advance Notice of Methodological Changes for Calendar Year (CY) 2021 for Medicare Advantage (MA) Capitation Rates, Part C and Part D Payment Policies - Part II (cms.gov)](https://www.cms.gov/files/document/2021-advance-notice-part-ii.pdf) (Accessed March 5, 2024); [↑](#footnote-ref-6)
7. [Final-2021-Benefit-Year-Final-HHS-Risk-Adjustment-Model-Coefficients.pdf](file:///C:\Users\kathl\OneDrive\Desktop\Project%20Santa%20Fe\CKD\Documents%20on%20HCC%20scores\Final-2021-Benefit-Year-Final-HHS-Risk-Adjustment-Model-Coefficients.pdf) (Accessed March 5, 2024). [↑](#footnote-ref-7)
